# Diazepam and Fluoxetine Decrease the Stress Response in Zebrafish

**DOI:** 10.1371/journal.pone.0103232

**Published:** 2014-07-23

**Authors:** Murilo Sander de Abreu, Gessi Koakoski, Daiane Ferreira, Thiago Acosta Oliveira, João Gabriel Santos da Rosa, Darlan Gusso, Ana Cristina Varrone Giacomini, Angelo Luis Piato, Leonardo José Gil Barcellos

**Affiliations:** 1 Programa de Pós-Graduação em Farmacologia, Universidade Federal de Santa Maria (UFSM), Santa Maria, RS, Brasil; 2 Universidade de Passo Fundo (UPF), Passo Fundo, RS, Brasil; 3 Departamento de Farmacologia, Instituto de Ciências Básicas da Saúde, Universidade Federal do Rio Grande do Sul, Porto Alegre, RS, Brasil; 4 Programa de Pós-Graduação em Bioexperimentação, Universidade de Passo Fundo (UPF), Passo Fundo, RS, Brasil; University of North Carolina at Chapel Hill, United States of America

## Abstract

The presence of pharmaceutical products in the aquatic environment has been reported in several studies. However, the impact of these drugs on living organisms is still uncharacterized. Here, we investigated the effects of acute exposure to either diazepam or fluoxetine on the stress response in *Danio rerio*. We showed that diazepam and fluoxetine inhibited the stress axis in zebrafish. Intermediate concentrations of diazepam suppressed the stress response as measured by cortisol levels, whereas fluoxetine inhibited cortisol increase at concentrations similar to those found in the environment. These data suggest that the presence of psychoactive drugs in aquatic ecosystems could cause neuroendocrine dysfunction in fish.

## Introduction

The presence of pharmaceutical drugs in the aquatic environment is a significant concern to regulatory agencies because these drugs could affect both the human population and aquatic ecosystems. Benzodiazepines and selective serotonin reuptake inhibitors (SSRIs) are present in wastewater effluent and are neither cleared nor photobleached after treatment of the effluents [1]–[3]. The environmental concentrations of these drugs range from 0.04 to 0.88 µg/L for diazepam [4]–[6] and 0.012 to 1 µg/L for fluoxetine [4], [7]–[10]. Benzodiazepines and SSRIs exert anxiolytic effects and can interfere with neuroendocrine stress axis activity [11]–[14]. Although these drugs have been detected in an extensive variety of environments, there is little information regarding the effects of these compounds in living organisms [15].

The stress response system helps the individuals to deal with adverse conditions [16]. For instance, increases in cortisol levels during stress can lead to hyperglycemia, which could provide energy for defensive actions [17]–[19], and also participate of the osmoregulation processes in fish [20]. Thus, the harmful effects of pollutants on the fish stress response can adversely affect their survival [21]–[22], since both drugs can interfere with stress response in humans [23]–[25]. In this context, we hypothesized that the concentrations of diazepam and fluoxetine in the environment can interfere with the stress response in fish. We tested this possibility using zebrafish (*Danio rerio*) as the experimental model. This fish species has many advantages as a model organism because of its easy handling and maintenance as well as its genetic homology with humans [26]–[28]. Recent studies have reinforced the use of the zebrafish model for stress research [28]–[35].

## Materials and Methods

### Ethical note

This study was approved by the Ethics Commission for Animal Use (CEUA) at Universidade de Passo Fundo, UPF, Passo Fundo, RS, Brazil (Protocol *#*7/2013-CEUA) and met the guidelines of Conselho Nacional de Controle de Experimentação Animal (CONCEA).

### Animals

A stock population of 1188 mixed-sex, adult wild-type zebrafish (*Danio rerio*) of the short-fin (SF) strain were held in 2 tanks with constant aeration and equipped with biological filtering under a natural photoperiod (approximately 14 h light: 10 h dark). Water was maintained at 26±2°C and pH 7.0±0.25, with dissolved oxygen levels at 6.5±0.4 mg/L, total ammonia levels at 0.01 mg/L, total hardness at 6 mg/L, and alkalinity at 22 mg/L CaCO_3_.

### Experimental design

**Figure 1:**
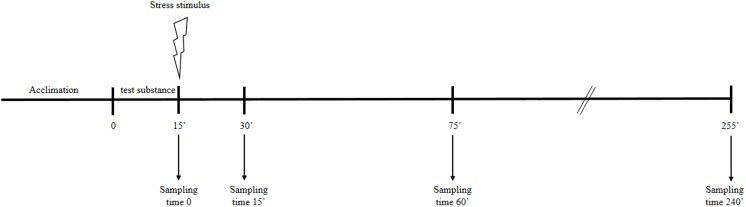
Schematic view of the experimental design of the study. The test substance refers to the control (no substance), diazepam (0.88, 16, or 160 µg/L) or fluoxetine (1, 25 or 50 µg/L).

For each test substance (fluoxetine or diazepam), fish from the stock population were distributed in 32 glass aquaria (30×30×30 cm, six fish per tank), acclimatized for seven days and fed with commercial food flakes (TetraMin, Tetra, Melle, Germany). Twenty-four hours later, fish were exposed to the test substance for 15 minutes. Animals were then submitted to a stress stimulus, consisting of chasing fish with a net for two minutes was applied [30], and sampled after 0, 15, 60 and 240 minutes for whole body cortisol analysis (Fig. 1). Similarly, groups were submitted to test substance without stress test (sampled at the same time points), aiming to evaluate an eventual stress effect of the substance per se. A basal situation, i.e. without drug exposure and stress test was performed as control.

This setup was replicated 3 times. For whole-body cortisol determination, pools of 2 fish (to obtain approximately 0.5 g of tissue) were examined, with a total of 6 pools of 2 fish for each treatment and time point.

Diazepam (União Química, 5 mg/ml) was used at the following three concentrations: 0.88 µg/L, which is the highest detected environmental concentration [5]; 16 µg/L, which is 10% of the concentration that promotes behavioral effects; and 160 µg/L, which is the concentration with reported effects in zebrafish behavior [36].

Fluoxetine (Daforin EMS, oral solution, 20 mg/mL) was tested at concentrations of 1 µg/L [7], 25 µg/L and 50 µg/L (25 and 50 times the environmental concentration, respectively).

### Cortisol extraction and analysis

Fish were captured and immediately frozen in liquid nitrogen for 10–30 s, followed by storage at −20°C until cortisol extraction. Whole-body cortisol was extracted using the method described by Oliveira et al. [30]. The accuracy was tested by calculating the recoveries from samples spiked with known amounts of cortisol (50, 25 and 12.5 ng/mL). The mean detection of spiked samples was 94.3%. All of the cortisol values were adjusted for recovery with the following equation: cortisol value = measured value × 1.0604.

Tissue extracts were resuspended in 1 mL PBS, and whole-body cortisol levels were measured in duplicate for each extraction using a commercially available enzyme-linked immunosorbent assay kit (EIAgen CORTISOL test, BioChem ImmunoSystems). This kit was fully validated for zebrafish tissue extracts using the methodology described by Sink et al. [37]. Precision was tested by performing 12 repeated assays on seven randomly chosen samples on the same 96-weel plate and calculating the intra-assay coefficient of variation (CV). Reproducibility was tested by assaying the same samples on different plates and calculating the inter-assay CV. To test for linearity and parallelism, the tissue extracts underwent serial dilutions in the buffer provided with the kit. A strong positive correlation between the curves was observed (R^2^ = 0.9108), and it was determined that the samples had low inter- and intra-assay CV values (7–10% and 5–9%, respectively).

### Statistics

Homogeneity of variance was determined using Hartley's test, and normality was determined using the Kolmogorov-Smirnov test. Whole-body cortisol concentrations were compared using a two-way ANOVA, with drug concentration and time after the stressor as the independent variables, followed by a Bonferroni post-test. Differences with p values <0.05 were considered to be statistically significant.

## Results

**Figure 2:**
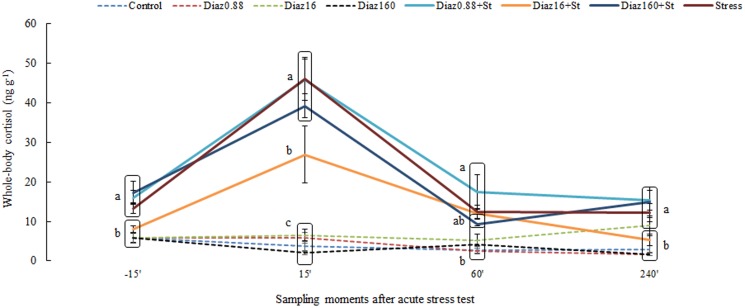
Whole-body cortisol concentrations in zebrafish to diazepam followed by an acute stress test and respective controls. The values are expressed as the mean ± standard error of mean. Different small letters indicate significant group differences in each sampling time.

There were a significant interaction between drug concentration and time after stress induction (P<0.0001; F_3,21_ = 9.086). Fish exposed to 16 µg/L of diazepam and submitted to the acute stress test (Fig. 2) had a reduced cortisol response to an acute stressor at 15 and 240 minutes after stress. Any effect on cortisol profiles were detected on the time course curve, in fish exposed only to diazepam without acute stress test.

**Figure 3:**
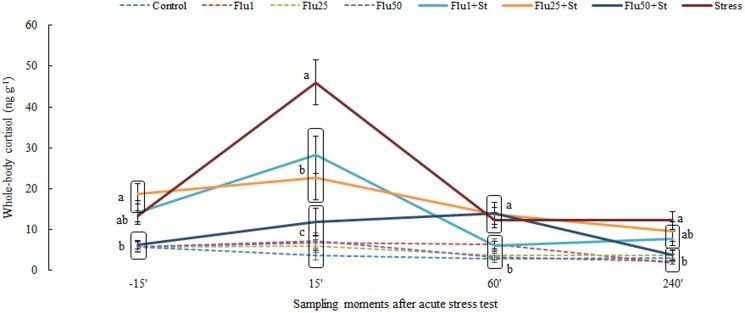
Whole-body cortisol concentrations in zebrafish to fluoxetine followed by an acute stress test and respective controls. The values are expressed as the mean ± standard error of mean. Different small letters indicate significant group differences in each sampling time.

For fluoxetine exposure (Fig. 3), we found a significant interaction between drug concentration and time after stress induction (P<0.0001; F_3,21_ = 7.492). Exposure only to the three fluoxetine concentrations did not cause any effect on whole-body cortisol while the combination of drug exposure and stress test resulted in an impaired cortisol response to an acute stressor at 15 minutes compared to the stressed only fish, with the highest concentration (50 µg/L) causing the strongest inhibitory effect. However, these fish (Flu 50 µg/L+stress group) had lower pre-stress concentrations compared to groups Flu 1 µg/L+stress and 25 µg/L+stress.

## Discussion

We show that acute exposure to diazepam and fluoxetine diluted in water impair the stress axis function, as drug-exposed fish had lower cortisol levels than control fish when exposed to an acute stress test. Both benzodiazepines and SSRIs possess anxiolytic activity [2], [14], [25], [36], and it is reasonable to suggest that these deleterious effects on the stress axis were related to a central action of these drugs.

The mechanism by which these drugs reduce the stress response may be directly related to the hypothalamus and/or pituitary gland, likely without the involvement of interrenal tissue. The serotonergic system of the brain plays a key role in autonomic, neuroendocrine and behavioral integration of the stress response in fish as well as mammals [38]. Benzodiazepines have been shown to influence the activity of the hypothalamus-pituitary-adrenal axis in humans by reducing basal adrenocorticotropic hormone (ACTH) and cortisol release after acute administration [39]–[41].

Our results showed that only the intermediate concentration of diazepam (16 µg/L) altered cortisol levels after stress induction. Other concentrations did not affect the cortisol stress response, demonstrating that the concentration of diazepam in the environment [5] was insufficient to impair the stress axis. Surprisingly, the highest concentration used (160 µg/L) did not alter cortisol response, suggesting diazepam may have a U-shaped dose-response curve similar to the effects of alcohol on fish behavior [30], [39] and stress response [42]. Similar curve pattern was found in cortisol effects on human memory [43].

However, acute exposure to all of the tested concentrations of fluoxetine impaired the cortisol response to acute stress, including the concentration identified in the environment (1 µg/L).

Although the anxiolytic effect exerted by diazepam and fluoxetine is well understood, the exact mechanisms by which these drugs block the biological response of cortisol in response to stress are still unclear. A fish with an impaired stress response loses its ability to maintain homeostasis against stressors by reducing the ability to promote ionic, metabolic and behavioral adjustments necessary for the stress response [22], [31]–[33]. A fish unable to display a normal cortisol response shows a reduced ability to respond to ongoing challenges posed by aquaculture [40]. Additionally, increased activity and boldness as well as reduced sociability in zebrafish that came in contact with these substances may also increase the risk of predation [41], [44], making the consequences of environmental contamination with these drugs difficult to predict.

The effects of diazepam and fluoxetine on cortisol levels have been previously reported in other species. For example, diazepam has been shown to decrease plasma cortisol levels, especially in humans experiencing stress [45]. Another study in elephant seals (*Mirounga angustirostris*) found that when animals were sedated with a combination of drugs ([tiletamine hydrochloride 50 mg/mL, zolazepam hydrochloride 50 mg/mL], ketamine, and diazepam) in the field, they did not exhibit a cortisol stress response. The use of this drug combination appears to decrease the responsiveness of the animals to handling and subsequent stress [46]. A similar result was observed in a study with Weddell seals (*Leptonychotes weddellii*), as treatment with diazepam improved the cortisol response of handled animals [47]. With regard to fluoxetine, chronic exposure to this drug decreased the cortisol levels in zebrafish in the novel tank test [28], [29]. Therefore, despite the differences in protocols and models, we have shown for the first time that animals exposed to either diazepam or fluoxetine diluted in water have a blunted cortisol response when exposed to a highly stressful situation.

Our results highlight that the presence of anxiolytic drugs in aquatic ecosystems may promote ecologically important but undervalued effects. It is imperative that new testing protocols be developed to examine the environmental impact of waste pharmaceuticals on fish and other aquatic life. Together with previous research, our data indicate that zebrafish are sufficient model organisms for studying pharmaceutical pollutants and their impact on the environment.
